# Moving Toward Individual-Specific Automotive Seat Design: How Individual Characteristics and Time Alter the Selected Lumbar Support Prominence

**DOI:** 10.1177/00187208211042776

**Published:** 2021-09-27

**Authors:** Jessa M. Buchman-Pearle, Kayla M. Fewster, Brendan L. Pinto, Jack P. Callaghan

**Affiliations:** 1 8430 University of Waterloo, Ontario, Canada; 2 University of British Columbia, Vancouver, Canada

**Keywords:** prolonged driving, low back pain, spine biomechanics, sex

## Abstract

**Objective:**

To explore how individual characteristics influence selected lumbar support prominence (LSP), seated lumbar flexion, seatback average pressure, contact area, and center of pressure (CoP) location before and after 1 hr of driving.

**Background:**

An LSP can alter posture and may reduce low back pain during prolonged driving. Although LSP preference varies across individuals and may change over time, few investigations have explored the time-varying response to individually selected adjustable seat parameters.

**Method:**

Forty individuals selected LSP settings in an automotive seat through a series of systematic adjustment trials. The average LSP setting was fixed for a 1-hr driving simulation, followed by one final adjustment trial. Regressions were performed between individual characteristics and selected LSP, lumbar posture, and measures of seatback pressure from the initial adjustment trials. ANOVAs were performed to determine the effect of time and sex on these dependent variables. Discomfort was also monitored throughout the protocol.

**Results:**

Individual’s standing lumbar lordosis, selected LSP, and height and mass were significant predictors for seated lumbar flexion, seatback average pressure, and contact area, respectively. Discomfort levels remained low; however, following the driving protocol, individuals altered their posture to decrease lumbar flexion and increase seatback average pressure without significant adjustments to the LSP.

**Conclusion:**

These findings highlight individual characteristics to consider in automotive seat design and that the method for determining LSP settings may facilitate appropriate LSP selection.

**Application:**

A systematic method to determine LSP settings may reduce discomfort and automate seat adjustments, such that only short-term postural adjustments may be required.

## Introduction

There is an established association between low back pain development and time spent driving (Alperovitch-Najenson et al., 2010; Anderson, 1992; Bovenzi, 2010; Chen et al., 2005; Gyi & Porter, 1998; Mendelek et al., 2011; Okunribido et al., 2007; Plouvier et al., 2008; Porter & Gyi, 2002; Robb & Mansfield, 2007), likely in part due to prolonged exposure to sitting. Compared to standing, sitting leads to flexion of the lumbar spine, resulting in posterior pelvic rotation and loss of the lumbar lordosis (De Carvalho et al., 2010; Endo et al., 2012; Keegan, 1953). This increase in lumbar flexion is linked to increased intradiscal pressure (Adams & Hutton, 1985; Andersson et al., 1974; Wilke et al., 1999), posterior migration of the nucleus pulposus (Alexander et al., 2007), increased strain on passive tissues (Dunk & Callaghan, 2002; McGill & Brown, 1992), and altered erector muscle fiber orientation (Pinto et al., 2021). These biomechanical and physiological adaptations may be further exacerbated during prolonged sitting as progressive deformation of the seat and viscoelastic creep of soft tissues occur. Moreover, in driving, the inability to vary posture due to task constraints (i.e., maintain gaze on road, feet on pedals, hands on steering wheel, and wearing a seatbelt) coupled with vibration exposure may further increase the risk of developing low back pain (Bovenzi, 2010; Porter & Gyi, 2002; Wilder et al., 1982). As such, there is a need to modify automotive design to reduce potentially maladaptive changes to spinal health and discomfort associated with prolonged driving.

Adjusting the lumbar support in automotive seats represents a feasible and effective approach to improve user comfort. The implementation of a lumbar support has decreased lumbar flexion (Andersson et al., 1979; De Carvalho & Callaghan, 2012; Lin et al., 2006; Makhsous et al., 2003), increased intervertebral disc height and reduced disc pressure (Andersson et al., 1974; Makhsous et al., 2003), and decreased muscle activity (Andersson et al., 1974; Kingma & van Dieën, 2009; Makhsous et al., 2009, 2003). Additionally, there are reports of decreased low back pain in drivers who utilize a lumbar support (Chen et al., 2005; Porter & Gyi, 2002). Unfortunately, when the lumbar support does not meet the user’s preference, possibly because it does not conform to the lumbar curvature, discomfort may occur (De Carvalho & Callaghan, 2015; Reed & Schneider, 1996). Recommendations for lumbar support settings have therefore been made to accommodate multiple users. The deflection of the lumbar support, lumbar support prominence (LSP), with a horizontal excursion of 2–5 cm measured with a standardized approach and calibrated manikin (Roe et al., 1999; Schneider et al., 1985, Schneider et al., 1985) at the spinal L2–L5 level (De Carvalho & Callaghan, 2015; Reed et al., 1994), has been suggested. However, sex differences in selected LSP excursion have also been noted, whereby females preferred greater excursion (average 3.25 ± .71 cm) than males (average 2.56 ± .88 cm; De Carvalho & Callaghan, 2015). With that said, the individual factors underlying these sex differences, such as anthropometric variability or previously observed differences in lumbar lordosis (Callaghan et al., 2010; De Carvalho et al., 2010; Endo et al., 2012), have largely been unexplored.

An additional limitation of current lumbar support investigations is that LSP excursion preference has only been measured over short-durations (e.g., 5–10 min [Lim et al., 2000; Reed & Schneider, 1996] or at the start of the driving protocol [De Carvalho & Callaghan, 2015]). Increasing lumbar flexion and posterior pelvic tilt were observed throughout a 1-hr simulated driving protocol (Callaghan et al., 2010; De Carvalho & Callaghan, 2011); thus, time-varying adjustments to lumbar support settings may assist in preventing these postural changes and associated discomfort. Interestingly, during a 2-hr driving protocol with a self-selected LSP excursion, no change in lumbopelvic posture was observed (De Carvalho & Callaghan, 2015); however, 76% of individuals indicated that they would have liked to adjust the LSP settings at some point during the drive (De Carvalho & Callaghan, 2015).

The aims of this study were twofold. The first objective was to explore how individual characteristics, including anthropometry, standing lumbar lordosis, and lumbar sagittal range of motion may influence self-selected LSP settings and automotive seat biophysical response. Individuals were first taken through a psychophysical method for determining LSP settings. Selected LSP excursion was recorded, and automotive seat biophysical response was quantified by seated lumbar flexion, seatback average pressure, seatback contact area, and height of the seatback center of pressure (CoP) above the midpoint of the iliac crests (i.e., approximate height of L4). We hypothesized that individual characteristics would moderately predict selected LSP excursion and measures of automotive seat biophysical response. The second objective of this study was to determine if selected LSP settings and automotive seat biophysical response were altered following a 1-hr driving protocol. It was hypothesized that following the 1-hr driving protocol, individuals would select a greater LSP excursion, and demonstrate decreased lumbar flexion, increased seatback average pressure and contact area, and a shift in height of the CoP toward the lumbar spine. In defining the relationship between individual characteristics and automotive seat biophysical response, as well as how responses may change over time, this research can build on the guiding ergonomic principle: to reduce pain and injury and improve productivity, the workstation (i.e., the automotive seat) must fit the individual.

## Methods

### Sample Population

Forty individuals were recruited from the university population. Individuals were included if they were licensed drivers and had no history of musculoskeletal injury or low back pain within the last 12 months. The project was approved by the University of Waterloo’s Research Ethics Board. Following providing written consent, age and anthropometric measures were recorded (Table 1).

**Table 1:** Mean and (Standard Deviation) of Sample Age and Anthropometric Measures

Parameter	Male (*N* = 22)		Female (*N* = 18)		Total (*N* = 40)	
Age (years)	23.7	(3.1)	21.9	(3.5)	22.9	(3.4)
Height (m)	1.77	(0.06)	1.62	(0.07)	1.70	(0.10)
Mass (kg)	81.1	(13.9)	61.1	(13.0)	72.1	(16.5)
BMI (kg/m^2^)	25.9	(3.5)	23.3	(3.8)	24.7	(3.8)
Waist circumference (cm)	86.1	(10.4)	71.5	(13.3)	79.5	(13.6)
Hip circumference (cm)	101.8	(7.3)	92.4	(15.1)	97.6	(12.1)

*Note*. BMI = body mass index.

### Instrumentation and Experimental Protocol

Prior to commencing any seated trials, a 5-s upright standing, maximum standing flexion, and maximum standing extension trial were performed for bias removal and normalization to maximum flexion for the accelerometer data and to determine lumbar spine range of motion.

**Figure 1:**
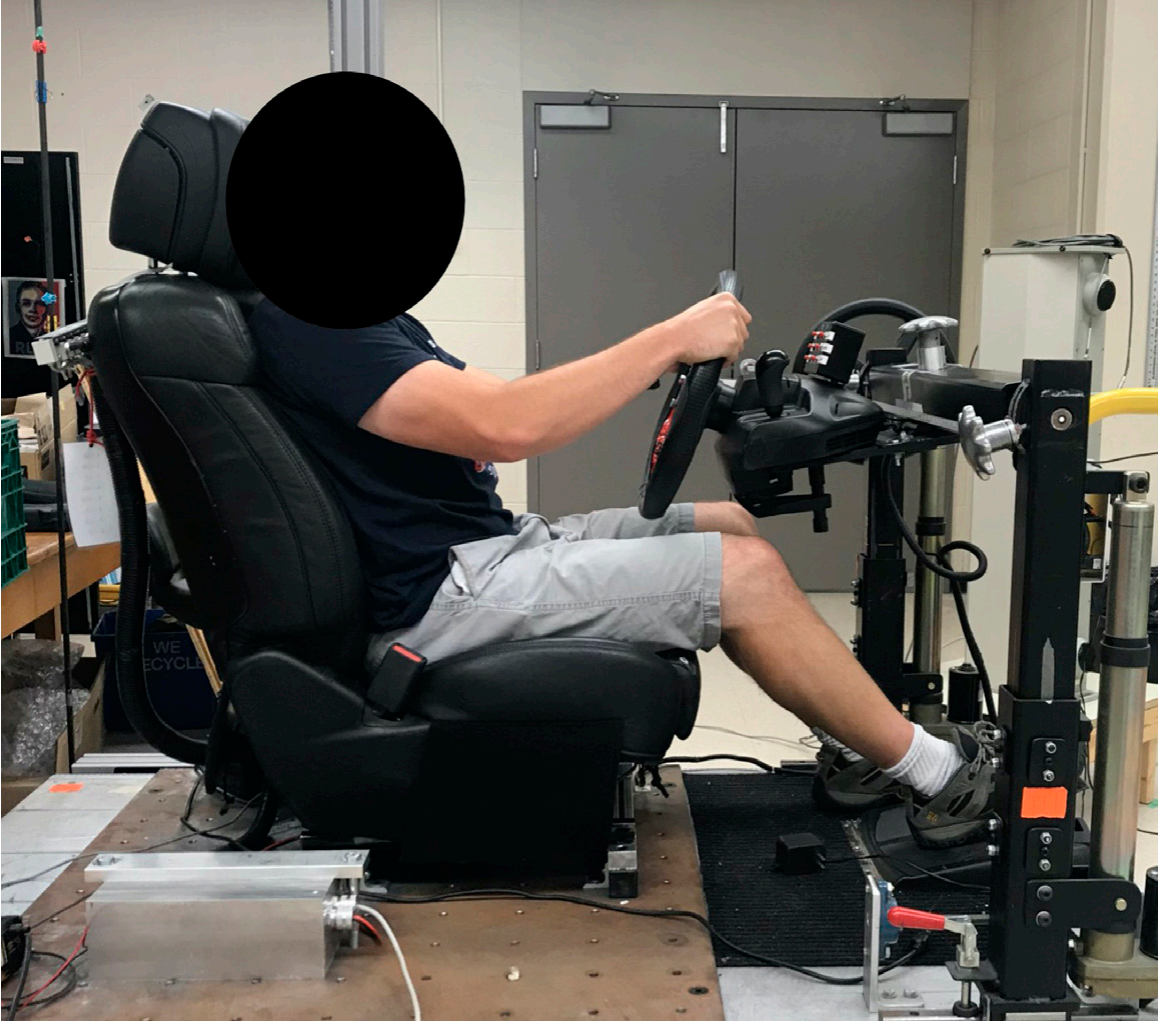
Individual seated in automotive driving simulator.

Individuals were then introduced to the driving simulator (STISIM Drive™ v2.0, Systems Technology Inc., Hawthorne, CA, USA), and the seat was adjusted to ensure consistency across the sample. Individuals were first seated in the driving simulator with the position of the automobile seat set such that a 110º knee angle was achieved. They were instructed to sit with their backs against the seatback, face forward, right foot on the gas pedal, hands on the steering wheel at the 10 and 2 o’clock positions, and to otherwise assume a normal seated driving position (Figure 1). All other adjustable features of the automobile seat, including the backrest angle/bolsters, and fore/aft position, were locked across the sample. The headrest was placed level with the top of the head and 5–10 cm back from the individual’s head (Insurance Bureau of Canada, 2002; Figure 1). Individuals were then familiarized with how to adjust the lumbar support settings by inflating three vertically stacked pneumatic bladders located in the seatback. LSP excursion was quantified using the current standardized SAE HPM-II (J4002) manikin and was measured in millimeters of horizontal shell deflection relative to a straight-back condition (Roe et al., 1999; Schneider et al., 1999).

The seatback was instrumented with a pressure mat (X3, XSensor Technology Corporation, Calgary, AB, Canada) to determine the pressure distribution. The pressure mat, which consisted of 1296 sensels (36 × 36) each with an area of 2.54 cm^2^, was placed such that the inferior row of sensels aligned with the seatback-seat pan boundary and was positioned to conform to the seatback curvature. To analyze the seatback pressure interface relative to the individuals, rigid marker clusters were adhered to the seat and sternum of the individual’s trunk (Optotrak, 3020/Certus, NDI, Waterloo, ON, Canada). To facilitate construction of a local coordinate system for the pressure mat, four points were digitized at the superior and inferior boundary of the backrest and bolsters on each side of the seatback, as well as a fifth point at sensel (1,1) on the pressure mat. Similarly, the right and left acromial processes and iliac crests were digitized and reconstructed relative to the marker cluster on the sternum. The individual was also instrumented with triaxial accelerometers (S2-10G-MF, NexGen, Montreal, QC, Canada) on T12/L1 and the sacrum of the pelvis, which acted as tilt sensors to measure motion of the lumbar spine. All digitized landmarks and motion tracking sensors were placed on the skin overlaying the bony landmark or segment. Pressure, motion capture, and accelerometer data were synchronously collected at 10, 50, and 100 Hz, respectively.

The experimental protocol consisted of (1) six seat-adjustment trials, (2) a 1-hr uninterrupted driving simulation, and (3) one seat-adjustment trial following driving. For the initial six adjustment trials, individuals were seated in the automotive seat and given 1 min to adjust the LSP settings. Once the settings had been chosen and the 1 min had elapsed, a 5-s trial was collected in the specified driving posture (Figure 1). The individual then stood up, the LSP settings were reset, and the next trial was initiated. The initial LSP settings were completed in a randomized order in which three trials began with the LSP fully disengaged (excursion of 9 mm) and down, and three trials began with the LSP fully engaged (excursion of 24 mm) and up.

The 1-hr driving protocol then commenced with the LSP set to the average LSP settings across the six trials. The driving simulator course was intended to represent highway driving and was projected onto a screen in front of the driver. Throughout the 1-hr driving protocol, individuals were instructed to keep both hands on the steering wheel at the 10 and 2 o’clock positions, stay on the simulation course, and maintain a constant speed of 100 km/h.

Ratings of perceived discomfort (RPDs) were completed at 15-min intervals throughout the prolonged driving trial for a total of five measures over the 1 hr. RPDs were collected bilaterally for the thoracic spine, lumbar spine, buttocks, and thighs with a 100-mm visual analog scale (VAS). Discomfort values were expressed relative to the baseline values collected before the experimental protocol. Following the driving protocol, individuals were permitted to adjust the LSP settings if desired, and a final 5-s trial was collected.

### Data Processing

Kinematic data, including both accelerometer and marker position data, were processed in a custom Matlab program (Version 2020b, Mathworks Inc., Natick, MA, USA). Gaps of up to .5 s in the marker position data were first filled with cubic spline interpolation; then both the motion capture and accelerometer data were dual-pass filtered with a low-pass second-order Butterworth filter with an effective cut-off frequency of 1 Hz. The voltage accelerometer data were converted into gravitational acceleration to determine the accelerometer orientation relative to gravity. The relative inclination between the accelerometers on T12/L1 and the sacrum were then used to determine lumbar spine flexion-extension angle in degrees. For the second study objective, investigating automotive seat biophysical response following a 1-hr driving protocol, the zero offset in upright standing was removed and the lumbar spine angle was normalized to maximum flexion (%MaxFlex). This normalization method could not be performed for the first study objective, as the lumbar angle in standing would have been zero for the whole sample.

Pressure data, including the average pressure (N/cm^2^), total contact area (cm^2^), and location of the CoP [sensel (row, column)] were exported from XSensor Pro (V7, XSensor Technology Corporation, Calgary, AB, Canada). The CoP vertical location was defined relative to the midpoint of the iliac crests. Any shift in the CoP would therefore be representative of a shift to or from the approximate location of L4 and reduce variability in CoP location simply due to an individual’s height. To accomplish this, a local coordinate system for the pressure mat was first constructed from the digitized points on the seatback with the origin defined at sensel (1,1). The location of the CoP in the pressure mat coordinate system was then transformed into the laboratory coordinate system with a local to global transformation. The vertical component of the vector from the midpoint of the iliac crests to the CoP was then calculated.

### Statistical Analysis

All statistical tests were performed in SPSS (SPSS, Version 20, IBM Corporation, Somers, NY, USA). Eight one-way repeated measures analyses of variance (ANOVAs) were performed on the RPD throughout the protocol (15, 30, 45, and 60 min) for the left and right thoracic spine, lumbar spine, buttocks, and thighs. When the ANOVA indicated a significant difference (*p* < .05), pairwise comparisons were performed with a Bonferroni-corrected alpha value.

Five dependent variables were defined: (1) selected LSP excursion, (2) seated lumbar flexion, (3) seatback average pressure, (4) seatback contact area, and (5) height of the seatback CoP relative the midpoint of the iliac crests. The average across the seat-adjustment trials (six pre- and one postdriving protocol) for each dependent variable were input into the statistical analyses.

Backward stepwise multiple linear regressions were performed to investigate the relationship between individual anthropometry and lumbar spine posture (independent variables) on the dependent variables (Babyak, 2004; Steyerberg et al., 2001). Independent variables were defined as: sex (0 for females and 1 for males), height, mass, body mass index (BMI), hip circumference, waist circumference, standing lumbar curvature, and lumbar spine range of motion. LSP excursion was also input as an independent variable into the regressions for the seated lumbar flexion, seatback average pressure, contact area, and height of CoP. Independent variables inclusion and exclusion criteria were set to *p* < .05 and *p >* .10, respectively. Hence, the stepwise process terminated when no independent variables remained or when all independent variables met the condition *p* < .10. Outliers were identified and removed if the measure for the dependent variable exceeded the sample mean plus or minus 3 standard deviations or an individual’s Mahalanobis distance exceeded the critical value calculated from the degrees of freedom for the final regression model. Each model was also assessed for collinearity (tolerance <.1 and variance inflation factor >5; Babyak, 2004), wherein stepwise regressions were performed by removing variables with the highest collinearity coefficients until all criteria were satisfied (Legendre & Legendre, 2012). The correlation coefficient (*R*), percentage of variation explained (*R^2^*), and level of significance, adjusted by a Bonferroni correction for the five regression models, were evaluated. Correlation coefficient strengths were defined using the following criteria: 0 to .3 as weak, .3 to .5 as low, .5 to .7 as moderate, and .7 to 1.0 as strong (Mukaka, 2012).

Five two-way mixed ANOVAs were also performed to determine the within-subject effect of time (pre- and post-seat-adjustment trials) and between-subject effect of sex (male and female) on each of the dependent variables. Pairwise comparisons were performed with a Bonferroni corrected alpha value when the ANOVA revealed a statistically significant difference (*p* < .05).

## Results

**Figure 2:**
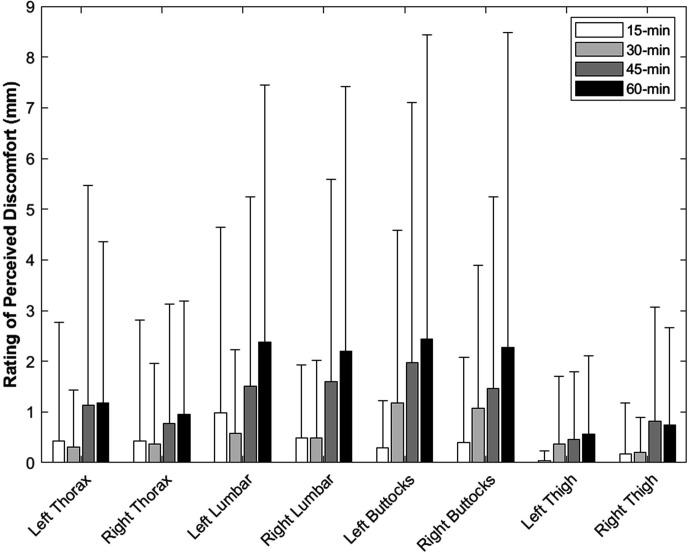
The mean and standard deviation (error bars) ratings of perceived discomfort (RPD), with baseline removed, for each body area throughout the driving protocol (15, 30, 45, and 60 min). The ANOVA revealed a main effect of time on RPD for the right lumbar spine and left buttocks, but the pairwise comparisons revealed no significant differences between any of the time points.

In general, the RPD remained low throughout the protocol with average scores for all body areas analyzed below 3 mm on the 100 mm VAS. Additionally, only 6 of 40 individuals reported a meaningful increase in low back pain (i.e., >8 mm [Hägg et al., 2003] for left or right lumbar spine) from baseline at least once during the protocol. The ANOVAs revealed a significant change in RPD for the right lumbar spine (*p* = .029, η^2^ = .094) and left buttocks (*p* = .015, η^2^ = .109); however, pairwise comparisons with a Bonferroni correction demonstrated no significant differences between time points (*p* > .060; Figure 2).

**Figure 3:**
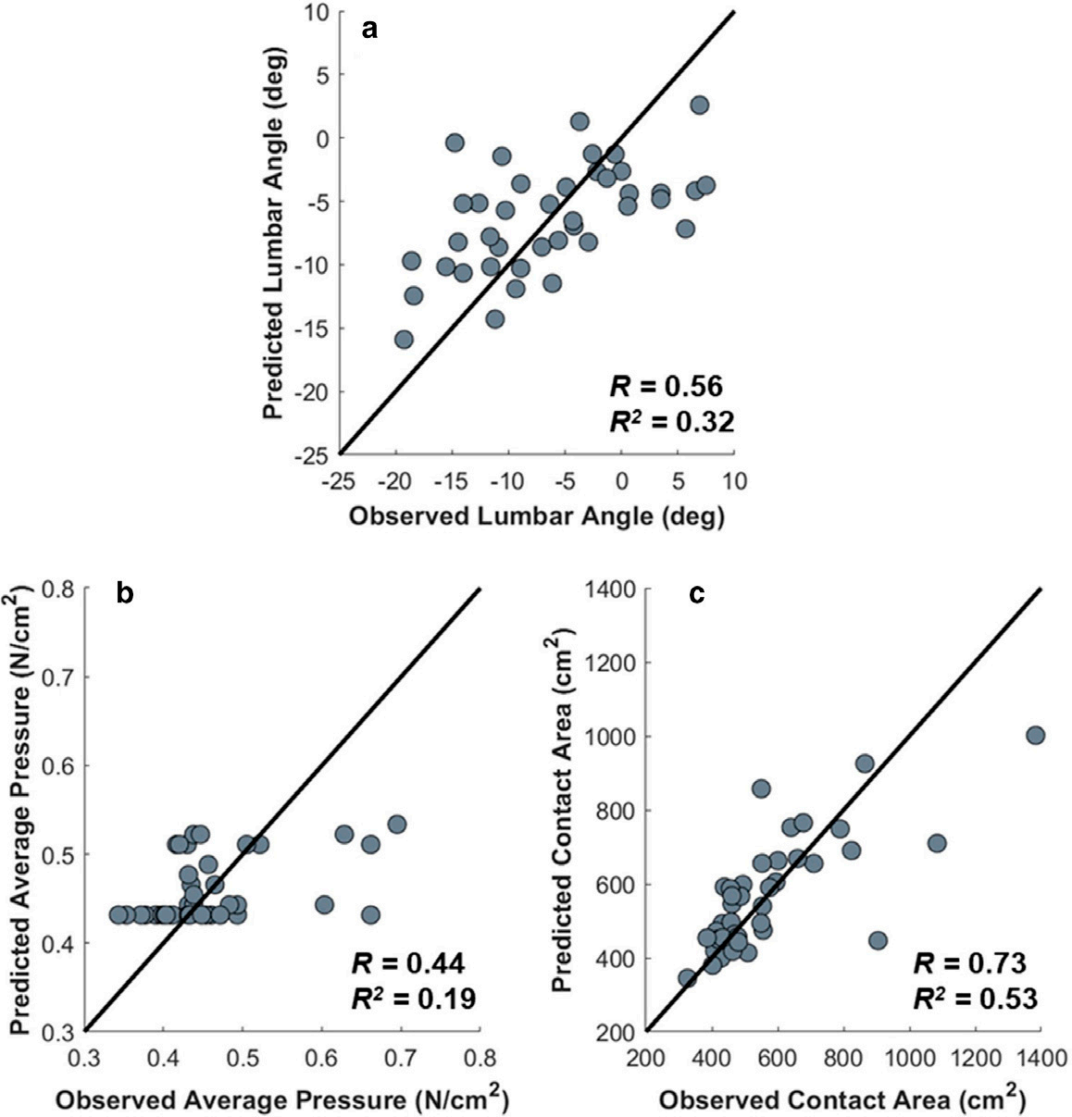
Observed versus predicted values for seated lumbar flexion (a), seatback average pressure (b), and seatback contact area (c). The *R* and *R^2^* for each model are indicated on each figure, and the black line indicates perfect agreement. For seated lumbar flexion, the lumbar angle refers to the inclination between the accelerometers on T12/L1 and the sacrum, where lumbar extension is more negative. For context, the average angle in standing was −28.8º and the average angle in sitting was −6.4º.

No individuals were identified as outliers in the regression analysis. Significant regression models were produced for the prediction of seated lumbar flexion, average seatback pressure, and seatback contact area from standing lordosis, LSP, and anthropometric factors, respectively (Figure 3). Model fit was defined as moderate for seated lumbar flexion (*R* = .56), strong for contact area (*R =* .73), and low for average seatback pressure (*R* = .44; Figure 3). The standing lumbar curvature explained 31.7% of the variance in seated lumbar flexion (*p* < .001; Eq. 1) and LSP explained 18.9% of the variance in the average seatback pressure (*p* = .025; Eq. 2). Together, individual height (partial *R* = −.35; *p* = .030) and mass (partial *R* = .68; *p* < .001) explained 53.2% of the variation in seatback contact area (*p* < .001; Eq. 3).

Eq.1 $$LL_{seated}=3.36+0.34\;LL_{stand}$$

Where $LL_{seated}$ is the seated lumbar lordosis angle (º) and $LL_{seated}$ is the standing lumbar lordosis angle (º).

Eq.2 $$P_{avg}=0.33+0.011\;LSP$$

Where *P_avg_* is the average seatback pressure (*N*/cm^2^) and LSP is the lumbar support prominence (mm).

Eq.3 CA=1051.85-799.37 height+12.18 mass

Where CA is the seatback contact area (cm^2^), height is the individual’s height (*m*), and mass is the individual’s mass (kg).

No significant model was produced for LSP excursion (*p* = .500), which consisted of individual mass (*p* = .036) and waist circumference (*p* = .058). Similarly, a significant model was not produced for height of the CoP (*p* = .060), which consisted of individual height (*p* = .063) and BMI (*p* = .098). Note that due to instrumentation error for the rigid marker cluster on the seatback, the height of the CoP could not be calculated for 9 of 40 individuals (*n* = 31; 16 female and 15 male).

**Figure 4:**
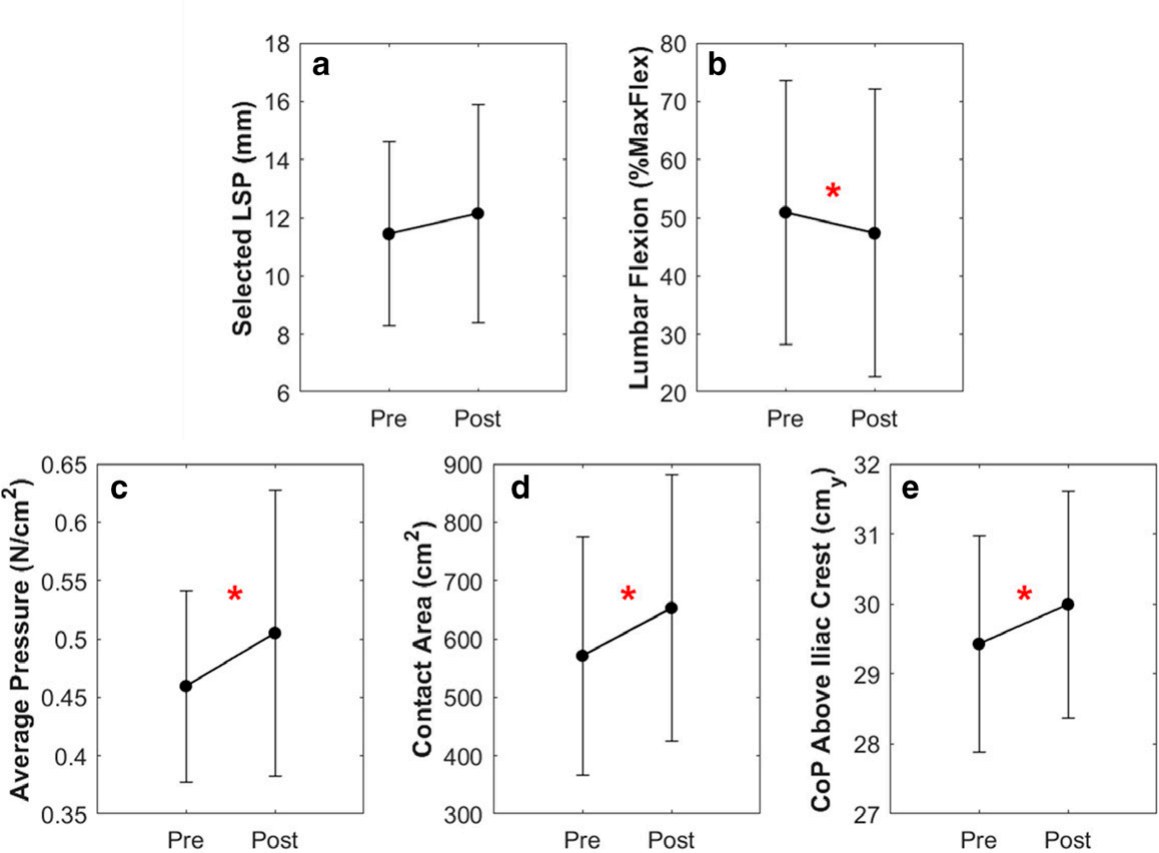
Pre- and post-1-hr driving simulation mean and standard deviation (error bars) for the seated lumbar lordosis (a), lumbar support prominence (LSP; b), average seatback pressure (c), seatback contact area (d), and center of pressure (CoP) vertical height above the midpoint of the iliac crests (e). The asterisks indicate a significant main effect of time (*p* < .05).

There was a significant main effect of time for seated lumbar flexion, seatback average pressure, contact area, and height of the CoP. Following the 1-hr driving protocol, individuals’ posture demonstrated an average decrease of 3.5% MaxFlex in lumbar flexion (*p* = .017, η^2^ = .14; Figure 4). The individual-seatback interface also demonstrated time dependent changes with an increase in the average pressure and contact area by an average .04 N/cm^2^ (*p* < .001, η^2^ = .34) and 47.2 cm^2^ (*p* < .001, η^2^ = .48; Figure 4), respectively. Individuals’ postural changes over the hour resulted in an average 5.7-mm increase in the height of the CoP away from the midpoint of the iliac crests (*p* < .001, η^2^ = .45; Figure 4). An average increase of .7 mm in the LSP pre- and postdriving protocol was not considered significant (*p* = .148, η^2^ = .05; Figure 4). As well, there was only a significant main effect of sex for seatback contact area (*p* = .038, η^2^ = .109), where contact area was 140.3 cm^2^ greater for males than females.

## Discussion

Individual characteristics predicted some of the variability in automotive biophysical response. Additionally, even without increases in RPDs throughout the driving protocol, individuals adjusted their posture to decrease lumbar flexion and increase measures of seatback pressure. As such, the first hypothesis was partially supported. Although a significant regression model was not produced for the selected LSP or height of the CoP, individual standing lumbar lordosis, selected LSP, and height and mass significantly predicted seated lumbar flexion, seatback average pressure, and contact area, respectively. The second hypothesis was also partially supported as individuals selected a posture that facilitated decreased lumbar flexion, and increased seatback average pressure and contact area; however, the height of the CoP increased away from the lumbar spine and no significant changes in LSP were observed. These findings highlight individual characteristics that should be considered in future automotive seat design and that the method utilized in the adjustment trials may facilitate consistent and appropriate LSP selection.

Given previously observed differences in LSP excursion preference across individuals (De Carvalho & Callaghan, 2015; Lim et al., 2000; Reed et al., 1994), it was surprising that individual characteristics were not significantly correlated with LSP. However, these findings can likely be explained by the overall low variability in LSP excursion across the sample, which only ranged from 9 to 18 mm prior to driving. Previous studies found that an LSP excursion of 10 mm reduced discomfort in a 10-min driving exposure (Lim et al., 2000) and that individuals selected an LSP excursion greater than 20 mm for a 1-hr driving protocol after exposure to five LSP excursions (0–5 cm; De Carvalho & Callaghan, 2015). In both investigations, the LSP was adjusted by the researcher at discrete intervals of at least 10 mm at a fixed vertical height; while, in the current study individuals were able to freely adjust the LSP excursion to a resolution of approximately 1-mm increments through three vertically stacked pneumatic bladders. This method of fine-tuning LSP excursion and vertical location may have further disguised individual differences for the dependent variables investigated. Nevertheless, the present findings indicate that an LSP excursion of approximately 10 mm may be recommended in automotive seats and is in the range of previously reported LSP magnitudes (De Carvalho & Callaghan, 2015; Lim et al., 2000)

Since the LSP settings were adjusted by each user, it was hypothesized that changes in LSP excursion would drive changes in posture and seatback pressure. Although there was no relationship between LSP excursion and seated lumbar flexion, likely because an LSP excursion of less than 20 mm at the L3 spinal level minimally alters the radiograph-determined lumbar lordosis in sitting (Andersson et al., 1979; De Carvalho & Callaghan, 2012), LSP alone explained 18.9% of the variance in the average seatback pressure (Figure 3). These findings suggest that even small modifications to LSP (<10 mm) may impact the user-seatback pressure interface. Multiple studies have explored the relationship between automotive seatback pressure and comfort (Kolich et al., 2004; Lim et al., 2000; Oudenhuijzen et al., 2003; Park et al., 2014). One study noted that an increase in LSP excursion above 10 mm (to 30 and 50 mm) resulted in significantly greater seatback pressure and an increase in discomfort (Lim et al., 2000). Due to the overall low discomfort in the current sample, the relationship between discomfort, LSP excursion, and seatback pressure could not be investigated. However, it is possible that increases in seatback pressure with an LSP could alter sensory feedback and perceived comfort, such that individuals feel more/less comfortable even in the absence of a changes to lumbar spine posture.

The regressions also revealed that seatback contact area and seated lumbar flexion were significantly predicted by individual height and mass, and standing lumbar lordosis, respectively. Unsurprisingly, seatback contact area was larger for taller individuals with a greater mass (Eq. 3) and larger for males. Alternatively, seated lumbar lordosis was moderately predicted by standing lumbar lordosis (*R* = .56). While individuals still demonstrated greater lumbar flexion in sitting than standing (Callaghan et al., 2010; De Carvalho et al., 2010; Endo et al., 2012), those with a greater lordosis in standing sat in a posture that facilitated decreased lumbar flexion (Eq. 1). These findings suggest that an individual’s standing lumbar lordosis may be an important parameter to consider in future automotive seat design. Further, the current findings imply that kinematic recommendations for a seated posture that mimics the lumbar spine curvature in standing (Keegan, 1953) may suffer from an inability to generalize to individuals with a standing lumbar lordosis beyond that of the sample investigated.

In addition to the potential influence of anthropometric and postural variability, the method for LSP selection can influence the posture adopted by an individual and the perceived comfort provided by a lumbar support. One study found that an individual’s seated lumbar lordosis was impacted more when the LSP excursion was adjusted with the user in the seat than when exiting/re-entering the seat between LSP excursion adjustments (Reed & Schneider, 1996). Thus, the LSP adjustments made by the individual within the adjustment trials may have more readily impacted their posture, despite the potential for less variability in their initial posture as individuals exited and re-entered the seat between trials. An additional study found that individuals would prefer minimal LSP excursion when first entering the seat, as the selected LSP was affected more by the available excursion range than the maximum available excursion (Kolich, 2009). Completing multiple adjustment trials with the initial LSP fully engaged or disengaged therefore maintained a relatively large LSP excursion range while exposing users to the minimum and maximum LSP excursion. To the authors’ knowledge, this is the first study to allow individuals to freely adjust the LSP settings, as discrete familiarization at different LSP excursions has typically been performed (De Carvalho & Callaghan, 2015; Lim et al., 2000; Reed & Schneider, 1996). Contrary to work by De Carvalho and Callaghan (2015), the current sample did not alter their LSP settings following the 1-hr driving simulation or report a meaningful increase in pain; therefore, the method herein likely facilitated appropriate individualized LSP selection and would be recommended for use in an occupational setting.

Despite no significant change in LSP following the driving simulation, individuals still adjusted their posture to reduce lumbar flexion, and increase seatback average pressure, contact area, and CoP height (Figure 4), indicating a tendency to lean further back into the seat. Previous studies indicate that individuals progressively flex and lean forward throughout prolonged driving (Callaghan et al., 2010; De Carvalho & Callaghan, 2011), which may negatively impact spinal health and increase discomfort (Callaghan et al., 2010; De Carvalho & Callaghan, 2011; Gruevski et al., 2016). The current analysis demonstrates that cueing postural adjustment, by providing individuals the opportunity to adjust their seat, was linked to postural changes which may combat previously observed increases in lumbar flexion. It is worth noting that the addition of an LSP alone maintained lumbopelvic posture throughout a 1-hr driving simulation; however, those individuals still reported an increase in discomfort (De Carvalho & Callaghan, 2015). The current sample adjusted their posture in the absence of meaningful increases in pain, further signifying the need for proactive adjustments to posture even with appropriate seat design.

The current study is not without its limitations. First, the LSP system did not enable excursion below 9 mm, which was the average selected LSP for 19 of 40 individuals. Since the initial LSP excursion can impact an individual’s perception of subsequent LSP excursion and comfort (Kolich, 2009), the selected LSP may have differed with a wider range of initial LSP settings. With that said, in a previous investigation where 0 mm of LSP excursion was tested, no individual preferred this excursion (De Carvalho & Callaghan, 2015). Second, although the current seat is commercially available and provides users with fine control of LSP settings, the vertically stacked bladder system may have masked relationships between independent/dependent variables and LSP excursion. For instance, individuals could have positioned the bladder system to conform to their natural lumbar curvature, thereby attenuating previously observed effects of sex on lumbar flexion in automotive seats (Callaghan et al., 2010; De Carvalho & Callaghan, 2011; Holmes et al., 2015). Moreover, the seat enabled users to select the same LSP excursion but at different vertical locations and unfortunately, the method of CoP localization may not have captured these small discrepancies in LSP vertical location. Future investigations may therefore benefit from more precise measurements of pressure distribution (Zehr et al., 2021) and lumbar support shape. Third, during collection, constraints were placed on elements of the automobile seat, including the backrest angle, fore/aft position, and the position of the hands on the wheel at 10 and 2 o’clock, which may not be representative of an individual’s preferred driving posture. However, controlling these factors facilitated explicit assessment of the LSP, the primary objective of this analysis. Lastly, young and healthy individuals with no history of low back pain within the past year were investigated. Given that kinematics and/or muscular demands vary with age (Gruevski & Callaghan, 2020) and pain status (Dankaerts et al., 2006; Dunk & Callaghan, 2010; O’Sullivan et al., 2006) when seated on stools or office chairs, the current results may be less generalizable to older individuals and/or those with previous low back pain, and assessment of these individuals in automotive seats is warranted.

Findings from this study demonstrate that automotive seat biophysical response varies as a function of individual characteristics and time. Correlations between standing lumbar lordosis and seated lumbar flexion, as well as height, mass, and contact area, suggest that these parameters should be considered in future automotive seat design. Additionally, the positive correlation between LSP and average seatback pressure may be linked to changes in sensory feedback and perceived comfort. Lastly, decreases in lumbar flexion and increases in seatback pressure without a significant change in LSP excursion or discomfort indicate that the proposed psychophysical method for seat-adjustment facilitates effective LSP selection across individuals. Utilizing this method may reduce the need for seat adjustments, wherein cueing short-term postural adjustments may be preferred.

## Key Points

- Individual standing lumbar lordosis, selected LSP, and height and mass were significantly associated with the seated lumbar flexion, seatback average pressure, and contact area, respectively.
- Following a 1-hr driving simulation, individuals chose a posture that facilitated decreased lumbar flexion, and increased seatback average pressure, contact area, and CoP height, despite no significant alteration to their selected LSP or pain development throughout the protocol.
- The method for selecting settings of an adjustable lumbar support appears to be an important consideration in individual-specific automotive seat design.
